# Drug-induced resistance: nipping it in the ‘budding’

**DOI:** 10.18632/oncotarget.26328

**Published:** 2018-11-13

**Authors:** Aaron Goldman

**Affiliations:** Aaron Goldman: Department of Medicine, Harvard Medical School, Boston, MA, USA; Division of Engineering in Medicine, Brigham and Women’s Hospital, Boston, MA, USA

**Keywords:** drug resistance, tumor heterogeneity, cell plasticity, DNA damage, combination therapy

Drug resistance remains one of the greatest hurdles towards successful cancer treatment, which underpins relapse, metastasis and mortality. Intrinsic features of resistance such as gene amplifications and over-expression of multi-drug resistance protein efflux pumps have been exhaustively studied to pin-point therapeutically tractable biomarkers, yet little progress has been made to thwart therapy failure in the clinic [1, 2]. An emerging concept in drug resistance is the role of adaptive and acquired mechanisms [3]. Islam *et al.* report on pages 35875-35890 new data in support of this latter concept, i.e. the role of drug-induced resistance *via* polyploidization in response to widely used anticancer therapies [4]. To study this phenomenon, they employed a model of high-grade Diffuse Large B-cell Lymphoma (DLBCL), time-lapse imaging, genomic, proteomic and kinomic profiling using patient-derived cells.

Polyploidization and aneuploidy are conserved phenomena in normal developmental biology that can contribute to metabolic proficiency and genetic diversification to resist exogenous stress [5]. But this protective, conserved evolutionary program can be co-opted by tumors to promote resistance under stress of environmental pressures such as anticancer cytotoxic and targeted kinase inhibitors of oncogenic pathways. Indeed, Islam *et al* previously reported that aneuploidy induced by aurora kinase (AK) inhibitors contributes to a survival phenotype in DLBCL [6]. In that report, they determined that an effective therapeutic strategy to mitigate the occurrence of aneuploidy after AK inhibition was the use of a three-hit drug combination comprising inhibitors of AK, BTK and CD20, which could significantly improve tumor growth inhibition [6]. Armed with the knowledge that this therapeutic intervention could prove challenging, the researchers set out to perform deeper molecular interrogations with the aim to discover novel tractable targets.

During a mechanistic investigation into the role of this polyploidization, Islam *et al* report several important observations. Firstly, they made use of time-lapse imaging to determine that a widely-applied combination of drugs including AK inhibitors with other cytotoxic agents result in a diversity of cell phenotypes. These include the induction of surviving 4n and >4n tumor cells, which undergo mitotic slippage, endoreduplication, and/or reductive cell division that could be described as ‘budding’ along with meiosis-type cell divisions leading to a cadre of variable aneuploid daughter cells capable of re-entering the cell cycle [4]. This makes sense because others, including our own lab, have pin-pointed rapid DNA amplification and cell state-switching as an escape mechanism in response to conventionally-used anticancer drugs [7]. Secondly, the researchers provide a molecular ‘view’ of these distinct phenotypic behavior using a multidimensional proteomic and transcriptomic screen integrated with a bioinformatic-based approach. They implicate a dynamic collaboration between Myc and Bcl2 with KPNA2, Ran-GAP1, TPX2 and AK-A as the molecular drivers of drug-induced phenotypic diversification and reductive cell division after relieving therapeutic pressure [4]. They further implicate gene ontologies associated with immune response, which could have major implications on combinations of drugs that target immune cells, such as checkpoint inhibitors.

Finally, Islam *et al* suggest novel therapeutic options to thwart drug-induced polyploidization and cell death-escape that can be integrated in combinatorial fashion. One argument they make is to target the enzyme Ran-GAP1, which functions as a lynchpin in their molecular cascade, while other drivers such as BCL-2 may only target a subset of the phenotypically-distinct drug resistant cells [4]. Thus, a rational combination and likely sequence or schedule of therapies is implicated in potential combination therapies. These novel therapeutic opportunities present greater complexity in targeting drug resistance. Indeed, we and others have suggested that a time-dependent administration of drugs can serve two purposes: 1) to reduce the proportion of tumor burden and ‘draw out’ phenotypically-transitioned drug resistant cells, and 2) administer a second drug in the combination to target the new vulnerabilities that are induced [8]. In this same way, the findings from Islam *et al* hint towards novel, temporally-sequenced anticancer drug combinations to improve clinical successes in high-grade DLBCL.
